# How to catch more prey with less effective traps: explaining the evolution of temporarily inactive traps in carnivorous pitcher plants

**DOI:** 10.1098/rspb.2014.2675

**Published:** 2015-02-22

**Authors:** Ulrike Bauer, Walter Federle, Hannes Seidel, T. Ulmar Grafe, Christos C. Ioannou

**Affiliations:** 1 School of Biological Sciences, University of Bristol, 24 Tyndall Avenue, Bristol BS8 1TQ, UK; 2 Department of Biology, Universiti Brunei Darussalam, Tungku Link, Gadong 1410, Brunei Darussalam; 3 Department of Zoology, University of Cambridge, Downing Street, Cambridge CB2 3EJ, UK; 4 Department of Animal Ecology and Tropical Biology, University of Würzburg, Am Hubland, Würzburg 97074, Germany

**Keywords:** *Nepenthes*, plant–insect interactions, carnivorous plants, prey capture, peristome ‘aquaplaning’, collective foraging

## Abstract

Carnivorous *Nepenthes* pitcher plants capture arthropods with specialized slippery surfaces. The key trapping surface, the pitcher rim (peristome), is highly slippery when wetted by rain, nectar or condensation, but not when dry. As natural selection should favour adaptations that maximize prey intake, the evolution of temporarily inactive traps seems paradoxical. Here, we show that intermittent trap deactivation promotes ‘batch captures' of ants. Prey surveys revealed that *N. rafflesiana* pitchers sporadically capture large numbers of ants from the same species. Continuous experimental wetting of the peristome increased the number of non-recruiting prey, but decreased the number of captured ants and shifted their trapping mode from batch to individual capture events. Ant recruitment was also lower to continuously wetted pitchers. Our experimental data fit a simple model that predicts that intermittent, wetness-based trap activation should allow safe access for ‘scout’ ants under dry conditions, thereby promoting recruitment and ultimately higher prey numbers. The peristome trapping mechanism may therefore represent an adaptation for capturing ants. The relatively rare batch capture events may particularly benefit larger plants with many pitchers. This explains why young plants of many *Nepenthes* species additionally employ wetness-independent, waxy trapping surfaces.

## 1. Introduction

Natural selection favours traits that increase the overall fitness of an organism [1]. Carnivorous plants rely on capturing animal prey to acquire crucial nutrients for growth and reproduction [2,3]. Strong selective pressures should act on their traps in order to maximize prey intake. Nevertheless, *Nepenthes* pitcher plants have evolved a temporarily ineffective trapping mechanism [4].

*Nepenthes* possess modified leaves (pitchers) that act as passive pitfall traps for (mainly) arthropod prey, enabling the plants to grow where essential nutrients (N and P) are scarce. Insects are attracted to the traps by visual and olfactory cues, and large quantities of sugary nectar are secreted at the inner margin of the pitcher rim (peristome) [2,5]. Slippery surfaces on the peristome and the inner pitcher wall cause visitors to fall into the pitcher and drown in the digestive fluid. In many species, the inner pitcher wall is covered with slippery wax crystals that may play a role in prey capture as well as retention [6–8].

While the wax crystals are effective at all times, the peristome is only slippery when wet. A combination of hydrophilic surface chemistry and micro-topography renders the peristome fully wettable. Under humid or wet conditions, thin and stable water films form on the surface, preventing the adhesive pads of arthropods from making full contact [9,10]. However, the peristome can be dry and safe to walk on for up to 8 h during the day. Variations in humidity and weather conditions act as a switch, intermittently activating and deactivating the trap [4].

The evolution of temporarily ineffective trapping surfaces seems paradoxical. Pitchers are costly structures that contribute little towards photosynthesis [11], and natural selection should favour mechanisms that maximize prey intake. Nevertheless, many *Nepenthes* species have reduced or lost the permanently slippery wax crystal layer and instead evolved larger peristomes [12]. The predominance of a temporarily inactive trapping mechanism in multiple, phylogenetically distant species suggests that this does not entail an overall disadvantage for the plant.

We previously hypothesized that the intermittent and unpredictable activation of *Nepenthes* traps facilitates ant recruitment and may represent a strategy to maximize prey capture [4,9]. In many pitcher plants, including our study species *N. rafflesiana*, ants are the predominant prey [13–15]. Ants are archetypal of collective behaviour where information is shared between individuals to the benefit of the whole colony [16]. ‘Scout’ ants explore new food sources and subsequently recruit nest-mates to exploit these [17]. Because of this scouting habit, temporary trap deactivation might not be a disadvantage as it has the potential to increase scout survival, ant recruitment and ultimately prey numbers. From this, we predict that constant trap activation by experimental wetting should have different effects on recruiting and non-recruiting prey. Constantly wet traps should capture more non-recruiting prey because of the extended active trapping time. For ants, we propose that this effect is annihilated or reversed due to the negative effect on recruitment under constantly wet (active) conditions. Moreover, we expect a shift in the mode of capture for ants: in the case of successful recruitment (under natural, intermittently wet conditions), ants should be captured in larger batches, whereas in the constantly wet case, they should be captured individually, similarly to non-recruiting insects.

## 2. Material and methods

Experiments were conducted in a secondary heath forest in Brunei (Borneo) during March–May 2008, June–July 2011 and May–June 2013. Our study species, *N. rafflesiana*, is the species for which the activation of traps by environmental wetness was originally described [4]. It grows in open, sunny habitats where daytime temperatures reach 37°C in the shade and the relative humidity drops below 50%, resulting in near-zero trapping efficiency during large parts of the day [4]. *Nepenthes rafflesiana* produces ‘lower’ pitchers on young rosette plants and ‘upper’ pitchers on mature climbing stems. ‘Upper’ pitchers lack wax crystals and depend mostly on the peristome for initial prey capture [18,19], while the pitcher fluid is important for prey retention [20]. *Nepenthes rafflesiana* pitchers capture a diverse range of insects, with ants dominating the prey spectrum (approx. 65% of captured individuals for upper pitchers). The remaining approximately 35% comprise mostly flying insects—Diptera, Hymnenoptera, Coleoptera and Lepidoptera are most common—along with a small number (<5%) of other arthropods such as spiders, termites and cockroaches [14,21].

### (a) Investigation of the natural capture mode

We investigated the frequency of batch capture events for one week by monitoring the natural prey intake of 43 (29 upper and 14 lower) pitchers on four individual plants with four to six shoots each. On each shoot, every functional pitcher was assigned a relative age (starting with ‘1’ for the youngest pitcher, ‘2’ for the next youngest, and so on). A pitcher was deemed functional when it was open, contained fluid and did not show major damage or drying in any of its parts.

Initially, all prey were removed and the fluid was filtered through a fine gauze mesh. A foam ear plug (Moldex-Metric, Nottingham, UK) was inserted into the tapered bottom end of each ‘upper’ pitcher to prevent prey from getting stuck and being lost to sampling. (‘Lower’ pitchers are ovoid in shape and did not need this treatment.) Prey were sampled twice in 3-day intervals by sucking out the pitcher contents with a 20 ml syringe with an attached silicon tube. All ants were sorted to morphospecies level and counted. Other prey items were identified at least to order, and to family or morphospecies where possible, and counted.

We calculated the index of dispersion, a quantitative measure of the distribution of captures across pitchers, for each prey taxon separately. The index of dispersion is defined as2.1
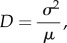
where *σ²* is the variance and *μ* is the mean number of prey per pitcher in a 3-day sampling period. *D* = 1 for randomly distributed, <1 for evenly distributed and >1 for aggregated (‘batch’) captures. 95% confidence intervals for *D* were estimated using bootstrap randomizations (*n* = 10 000). For rare prey classes, this often resulted in a mean and variance of zero during the bootstrap procedure, and hence an undefined index of dispersion. Prey classes where more than 5% of bootstrap resamples had an undefined index of dispersion were excluded from the results.

### (b) Comparison of continuously versus intermittently active traps

**Figure 1.**
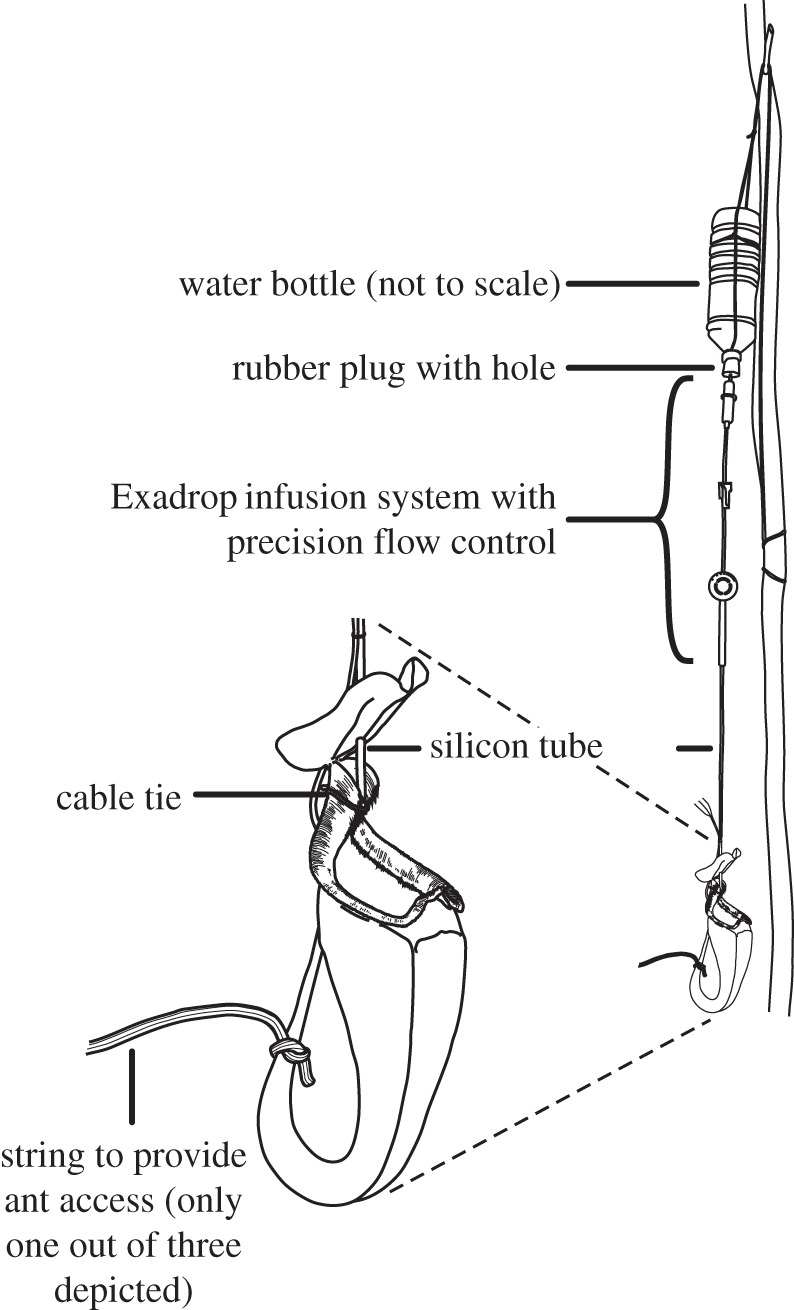
Experimental set-up to test the effect of constant wetting on ant recruitment and trapping. On each *N. rafflesiana* plant, two pitchers were prepared in this way but only one pitcher per pair was connected to the water supply. Wetted and control pitchers were swapped halfway through the experiment.

The effect of intermittent trap activation on prey capture was tested for pairs of ‘upper’ pitchers on 30 separate plants. Each pitcher was isolated from existing ant trails by applying a sticky resin (The Tanglefoot Company, Grand Rapids, MI) to the leaf base and reconnected to the surrounding vegetation via three pieces of string (cotton, 2 mm and 5 mm diameter; plastic, 2 mm diameter), allowing foraging ants to rediscover it. One pitcher of each pair was continuously wetted using an Exadrop infusion drip system (B. Braun, Melsungen, Germany) supplied with distilled water from a 1.5 l plastic bottle. The infusion tube was inserted through a hole in the pitcher lid and fixed with a plastic-coated wire tie (figure 1) so that water could spread evenly on the peristome. The untreated control pitchers were subject to natural fluctuations of environmental wetness. Pitchers were prepared for prey sampling as described above, and prey were sampled at least every other day for a total of 16 days. After 8 days, experimental and control pitchers of each pair were exchanged and the string connections renewed. Seven pairs had to be discarded due to pitcher damage during the experiment.

Ant and flying prey numbers were pooled across experimental periods for each condition (wetted and control). Chi-square tests were used to compare the prey totals. The effect of the wetting treatment on each individual pitcher was analysed using negative binomial generalized linear mixed models (GLMM). In addition to the experimental period, prey type (ant or flying), treatment (wetted or control) and the interaction of prey type with treatment were included as fixed factors. The sampled pitcher, nested in the plant, which was then nested in the experimental period, was used as a random factor. To examine the effect of the treatment on each prey type separately, this analysis (without prey type as a factor) was repeated for ants and flying prey individually. The significance of the interaction term and each main effect was determined by comparing models with and without the effect.

The effect of constant wetting on the frequency of batch captures was analysed separately for ants and flying prey by calculating the difference in the index of dispersion of prey captured by control versus constantly wetted pitchers, using the 2-day sampling intervals only. Two separate bootstrap randomizations (*n* = 10 000) were carried out: one to estimate the 95% confidence intervals for *D* for ants and flying prey in each treatment, and a second to determine whether the difference in *D* between wetted and control pitchers for each prey type was significantly greater than zero. GLMMs and randomization tests were carried out using the R v. 2.15.1 software package (R Foundation for Statistical Computing, Vienna, Austria).

To test the effect of constant wetting on ant recruitment, 19 individual pitchers of similar age, each on a different plant, were consecutively fitted with an infusion drip system, isolated from existing ant trails and reconnected to the surrounding vegetation in the same way as described above for the capture experiment. Immediately after this preparation, each pitcher was observed for 10 h with a digital camera (Pentax Optio W80, Ricoh Imaging Company, Tokyo, Japan) automatically taking an image every 5 min. Observations were started between 6.30 and 8.30. A different pitcher was observed each day, and we alternated between wetted (9 pitchers) and control (drip not switched on; 10 pitchers) from day to day. To ensure that observed differences in ant recruitment were not caused by dilution of the peristome nectar, we used 3% glucose solution for wetting. The images were analysed by counting ants and other visiting insects on the peristome separately. The numbers of ants and other insects, respectively, were pooled for each hour after allowing prey to access the pitcher and tested for an increase over time using Page's test in the software package BiAS for Windows (Epsilon Verlag, Frankfurt, Germany). Page's test is a non-parametric (i.e. rank-based) repeated-measure test that specifically looks for trends over time [22].

## 3. Results

### (a) Ants are commonly captured in batches

**Figure 2.**
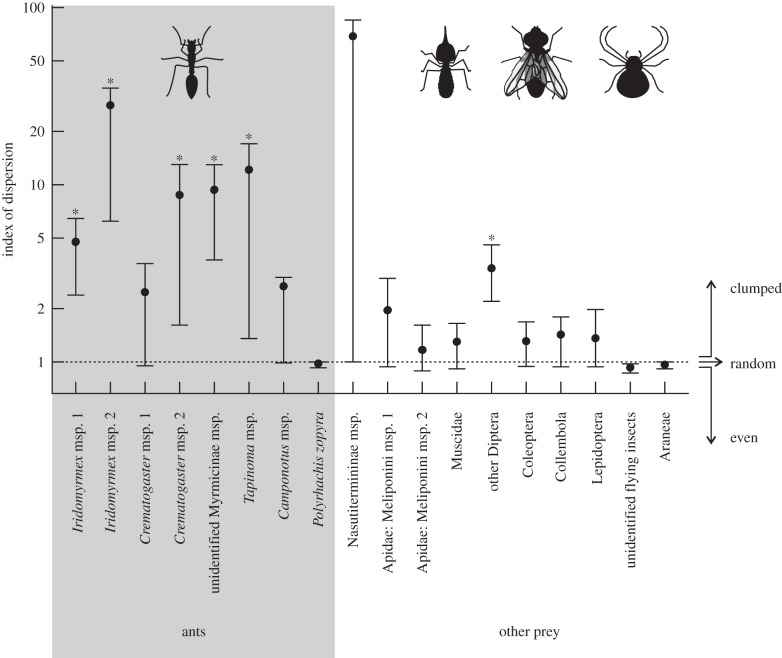
Index of dispersion for eight morphospecies of ants and three morphospecies and seven collective categories of other prey captured by *N. rafflesiana* pitchers in the field. The bars denote the 95% confidence interval for each index value estimated from bootstrap randomizations. Indices that are significantly above 1 (marked with an asterisk) indicate a clumped distribution, which is expected for batch capture events.

Despite the short sampling period for natural prey capture (2 × 3 days), we recorded multiple batch capture events for all four monitored plants. Batch captures of at least 20 individuals occurred in three plants, and the fourth captured a maximum of 19 individuals of one morphospecies. Most batch captures were ants (21 events ≥5, 10 events ≥10, 5 events ≥20 individuals) but termites were also occasionally represented (three batch capture events with 5, 48 and 86 individuals). We obtained indices of dispersion for eight morphospecies of ants, one morphospecies of termite, two morphospecies of stingless bees and seven higher taxonomic groups of non-hymenopteran insects and spiders (figure 2). For five out of eight ant morphospecies, the index of dispersion was significantly higher than one, indicating an aggregated distribution of capture events (i.e. batch captures). For two further ant morphospecies (*Crematogaster* msp. 1 and *Camponotus* msp.), the calculated indices of dispersion were 2.48 and 2.66, respectively, but the 95% confidence intervals marginally overlapped with one. The only ant species where only individuals were captured was *Polyrhachis zopyra*.

Termites, represented by a single morphospecies, had the highest index of dispersion (68.61) of all analysed taxa; however, the very large confidence interval just included one at the lower end. This probably reflects a lack of statistical power because of the rarity of termite captures within the short sampling period. Termites were only captured in four instances but two of these were large batch captures of 48 and 86 individuals, respectively.

The two morphospecies of stingless bees as well as all other prey groups (flies, beetles, springtails, moths, unidentified flying insect and spiders) were generally captured individually. The group of ‘other Diptera’ stands out with an index of dispersion significantly above one; however, this is unlikely to reflect batch captures as this specific category includes a diverse mix of morphospecies. While it was not unusual to find 10 or more individual small dipterans in a single pitcher sample, we hardly ever found more than three individuals of the same morphospecies.

The vast majority of batch capture events occurred in recently opened pitchers (electronic supplementary material, figure S1). Only the youngest pitchers on each shoot captured at least 10 ants of the same morphospecies within one sampling period, and still over 80% of all batch captures of at least 5 occurred in the youngest pitchers (Fisher–Freeman–Halton's exact contingency table test, d.f. = 2, *p* < 0.001 for batches ≥10 and ≥5, respectively). Batch captures also appeared to be slightly more frequent in ‘lower’ than in ‘upper’ pitchers (electronic supplementary material, table S2); however, the difference was not statistically significant (Fisher's exact test, *p* < 0.1 for batches ≥10 and 20, respectively, and *p* > 0.1 for batches ≥5).

### (b) Intermittent trap activation promotes batch captures of ants

**Figure 3.**
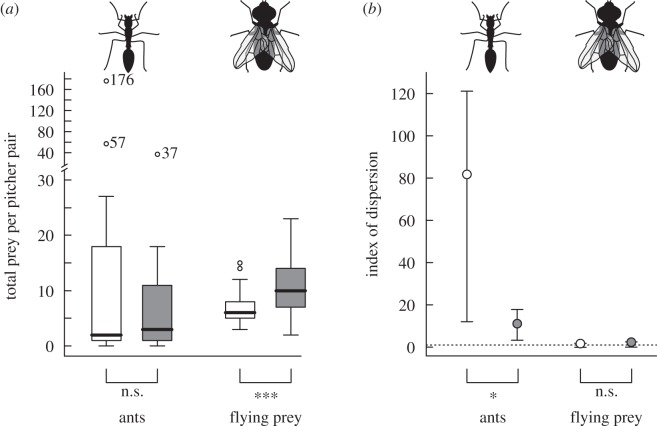
(*a*) Prey capture by natural (control, white boxes) and constantly active (wetted, grey boxes) pitchers within each plant. Bars denote medians, boxes represent the inner quartiles and whiskers contain all values within 1.5 times interquartile range. In the wetted case, more than 2/3 of all ants were captured by two individual pitcher pairs, rendering the 2.5-fold difference in across-pitcher ant prey sum non-significant on the individual pitcher level. Wetted pitchers captured on average significantly more flying prey. (*b*) Index of dispersion for prey captures by control (white circles) and wetted (grey circles) pitcher traps. The bars denote the 95% CI for each index value estimated from bootstrap randomisations. Ant captures were significantly more clumped in the control than in wetted pitchers.

In total, the intermittently active (control) pitchers captured 36.5% more prey over the course of the experiment. The sum of captured ants was significantly higher in the control group compared with the wetted treatment (339 versus 136 individuals, *χ*² = 86.76, d.f. = 1, *p* < 0.001), but the sum of all other prey was lower (151 versus 223 individuals, *χ*² = 13.86, d.f. = 1, *p* < 0.001). The GLMM analysis yielded a significant interaction between prey type and treatment (deviance_8,9_ = 5.49, *p* < 0.05). While the higher number of flying prey captured by wetted pitchers remained highly significant at the level of the individual pitcher (deviance_6,7_ = 13.22, *p* < 0.001), the difference in ant captures between the two experimental conditions was ‘concealed’ in the outlier range of the distribution (deviance_6,7_ = 0.006, *p* = 0.94; figure 3*a*). This suggests that the stark difference between the ant prey sums is entirely due to batch capture events.

The effect of batch captures is further confirmed by the index of dispersion. The difference in the distribution of captures between wetted and control pitchers was significantly greater than zero for ant prey (*p* < 0.05) but not for flying prey (*p* = 0.92; figure 3*b*). These results demonstrate that captures of ants by control pitchers occurred in more clumped, aggregated batches, in contrast to constantly wetted pitchers, and that this effect was not present for flying prey.

**Figure 4.**
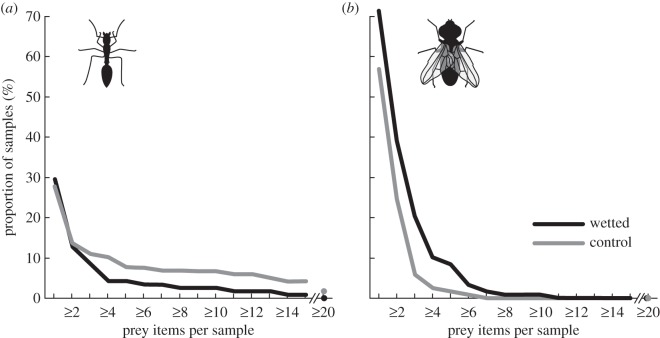
Effect of wetting on the capture of ant and flying insect prey. Samples were obtained in 2-day intervals. (*a*) Constant wetting decreased the frequency of batch captures of ants. (*b*) Constant wetting increased the number of flying prey captured. Irrespective of the treatment, flying insects were captured in low numbers.

Batch captures of ants were relatively rare events: approximately 90% of all 2-day samples (across both treatments) contained fewer than three ants. Large batches of ants (≥20 individuals over 2 days) were only ever captured by the control group, not by the wetted pitchers (figure 4*a*). Captures of 10 or more ants were more than twice as frequent in the control group (eight events) than in the constantly wet group (three events), and the difference is still notable for batches ≥5 (nine versus five events). For single ant captures, by contrast, we found no difference between control and wetted (33 versus 35 events). In line with what we found for the natural capture mode of pitchers, only ants were captured in batches, whereas flying insects were generally trapped in small numbers. The highest number of flying insects (of various species) captured over 2 days was 10, captured by a wetted pitcher. By contrast, the largest ant catches (both in the control group) contained 132 and 40 individuals, respectively.

Flying insects provided a reliable, steady but low intake of prey under both experimental conditions. Continuously wetted pitchers captured more flying insects, but still in an individual capture mode. This was confirmed by an increased proportion of pitchers that captured flying insects under constant wetting (71 versus 57%; Fisher's exact test, *p* < 0.05), while the number of captured individuals per pitcher per 2-day interval still remained relatively small: 89.9% of the wetted and 97.5% of the control samples contained three or fewer flying insects, and only five samples (four wetted, one control) contained more than five individuals (figure 4*b*). Hence, continuous wetting increased ‘baseline capture’ slightly (more pitchers captured low numbers of flying insects); however, under natural (control) conditions over 50% of all pitchers still captured at least one flying insect per 2-day period. Ants, by contrast, appeared less predictable as prey. Irrespective of the experimental conditions (wetted or not), 70% of all pitchers did not capture any ants within a 2-day sampling period.

### (c) Intermittent trap deactivation promotes ant recruitment

**Figure 5.**
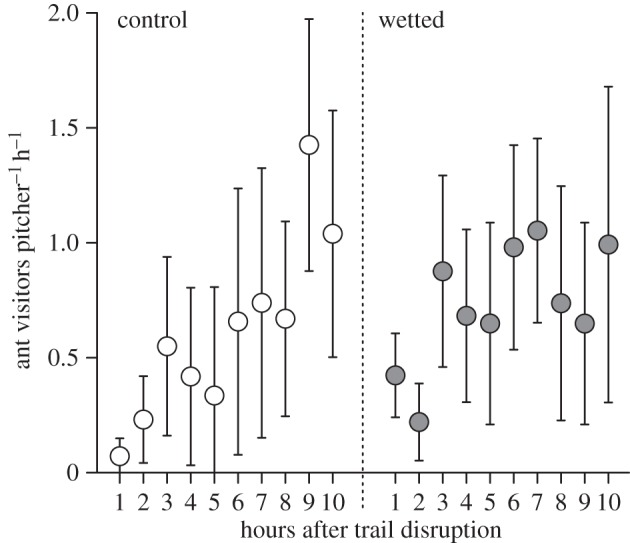
Discovery of and recruitment to natural (control) and constantly wetted pitchers by ants, observed with a time lapse camera (1 frame per 5 min) over a period of 10 h. The circles represent visitor numbers per hour (geometric means) and the whiskers denote ±1 s.e. Despite the high variability of ant visits between pitchers, a significant upward trend of hourly visitor numbers could be observed for control but not for wetted pitchers.

Despite pronounced variations of visitor numbers between pitchers and over time, the recruitment observations revealed a significant upward trend of hourly visitor numbers over time for ants visiting control pitchers (Page's test, *n* = 10 pitchers, 10 h, *L* = 3265.5, *p* < 0.01), but not for wetted pitchers (Page's test, *n* = 9 pitchers, 10 h, *L* = 2803.5, *p* = 0.16; figure 5) or other visitors to either group (*L* = 3006.0, *p* = 0.59 and *L* = 2634.5, *p* = 0.86). This indicates that ant recruitment was impeded by constant wetting.

## 4. Discussion

Our results show that temporarily ineffective pitcher traps do not represent a disadvantage for the plant. Our ‘scout hypothesis' predicted that a continuously highly effective trap is disadvantageous for capturing ants because it will kill a large proportion of scout ants, thereby hindering recruitment and depriving itself of subsequently increased visitor numbers. This was confirmed by the finding that constant trap activation (via artificial wetting) did not boost the number of captured ants but instead hindered recruitment and shifted the capture mode for ants from batch captures to individual captures.

### (a) Continuous versus intermittent trap activation: an optimality model

**Figure 6.**
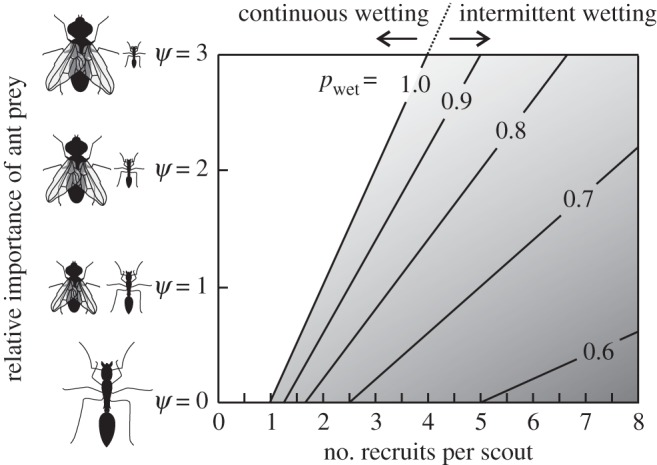
Predictions from a simple mathematical model (equation (4.1), plotted here for *E*_wet_ = 1) showing whether continuous or varying degrees of intermittent wetting maximize the pitchers' capture rate, depending on the recruitment rate of visiting ants and the relative importance of ants or other insects as prey. For example, if *ψ* = 1 (equal importance of ants and other prey) and if each ant recruits on average five further ants, the pitcher will catch the most prey if the peristome is wetted 70% of the time. *ψ* = nutritional importance of other prey compared to ants (for detailed definition see electronic supplementary material, appendix S3); *p*_wet_ = probability of the peristome being wet. Lower *p*_wet_ is favoured with increasing importance of ant prey and with higher recruitment rate.

A simple model for pitcher nutrient intake by insect trapping (see electronic supplementary material, appendix S3) confirms the potential advantages of intermittent wetting (or of a generally low capture rate) for the plant. Assuming that ant recruitment depends linearly on the number of recruiters [17] and that pitchers alternate between a high capture rate *E*_wet_ when wet and 0% when dry, the model predicts that intermittent wetting will maximize nutrient intake if4.1
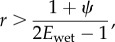
where *r* is the visiting ants' recruitment rate (visitors/time) and *ψ* (dimensionless) is the relative nutritional importance of other, non-recruiting prey compared to ants. Intermittent wetting is predicted to be optimal for pitchers if ants dominate the prey spectrum (low *ψ*) and if their recruitment is sufficiently efficient (high *r*), but continuous wetting would be favoured if ants are less abundant (high *ψ*) or less efficient recruiters (low *r*; figure 6).

Similarly, if the pitchers' capture rate is assumed to be constant, the model predicts that a *sub-maximal* (<100%) capture rate would maximize overall nutrient gain (consistent with previous predictions [23,24]) if *r* > (1 + *ψ*) (i.e. again if ants dominate the prey spectrum and if their recruitment is highly efficient). The model's conclusions hold for both linear and nonlinear (sigmoidal) recruitment, as long as the gradient of the recruitment curve is steep enough (see electronic supplementary material, appendix S3).

### (b) Mass capture of termites: recruitment or lucky strike?

The only other prey group that was occasionally captured in large numbers comprised termites. Similarly to ants, termites also show scouting and recruiting behaviour [25]; however, they do not feed on nectar and are therefore unlikely to recruit to *Nepenthes* pitchers. One notable exception is *N. albomarginata*, which attracts lichen-feeding *Hospitalitermes* and traps them in large numbers. The termites are attracted to a narrow ring of white trichomes on the pitcher outside, just underneath the peristome, and recruitment to this structure has been demonstrated for *H. bicolor* [26]. Interestingly, the location of the lichen-mimicking trichomes on the safe outside of the pitcher might also resemble an adaptation to promote scout survival. Other *Nepenthes* species (including the *N. rafflesiana* investigated here) have not been observed to attract termites. The occasionally observed mass capture events probably occur when migratory termite trails run across a pitcher by chance. This assumption is supported by the fact that we only found termites in pitchers growing at ground level and that termite migrations commonly take place at night [26,27] when the peristome is wet and slippery.

### (c) Risk or reliability: which is the better strategy?

How important are batch captures for the plant? After all, they are rare events: even in the control group, 90% of the samples contained five or fewer ants after 2 days (figure 4*a*). However, pitchers are functional traps for much longer than the 2-day sampling interval of our experiments. Moreover, when looking only at the youngest (and most trapping-active [21]) pitchers, 45% of the observed pitchers had captured five or more ants at least once in only two sampling intervals. *Nepenthes* plants can have dozens, sometimes hundreds of pitchers, and a batch capture event in any one of them will contribute significantly to the nutrition of the whole plant. In our experiment, even a low batch capture rate of 10% resulted in a 2.5-fold increase in overall ant captures across all 23 pitchers. We can therefore assume that, at least for large plants above a critical number of pitchers, it should pay off to pursue a strategy that promotes batch captures.

By contrast, small plants with fewer pitchers would play a risky lottery relying on batch captures and should pursue a more conservative trapping strategy that leads to a steady, low but more reliable prey intake. This might explain why in many *Nepenthes* species, we see a shift in trapping strategies from ‘lower’ to ‘upper’ pitchers. Seedlings start off growing as a rosette plant with relatively few, ‘lower’ type pitchers. In most species (including *N. rafflesiana*), these ‘lower’ pitchers have a slippery wax crystal coating on the inner wall surface, providing a continuously active, wetness-independent means of trapping. Because they have a peristome *in addition*, young plants also benefit from batch captures. Combining both strategies might help to maximize nutrient intake at a critical life cycle stage. When the plants mature, they grow climbing shoots with ‘upper’ pitchers that, in a large number of species, have lost or largely reduced the wax crystal cover [12,18,19]. This suggests that the production costs for wax crystals outweigh their benefits for prey capture in plants with a large number of pitchers.

### (d) Intermittent trap activation: an adaptation to exploit collective behaviour

The wetness-activated peristome might constitute a specialist adaptation for trapping ants. Intermittent trap activation by pitcher plants may have evolved to exploit recruitment behaviour of social insects that is otherwise highly beneficial to these organisms [28]. It has previously been suggested that a continually low capture success rate should be beneficial for trapping ants as it increases the probability for scout ants to survive [24]. We show here that a highly variable capture rate, alternating between ineffective and highly successful traps, can serve the same purpose. During dry times of the day, the nectar on the peristome dries up, presenting visiting scout ants with a highly concentrated, safely accessible sugar source. When the peristome gets wetted by condensation or rain, it turns extremely slippery and traps large numbers of visiting insects [4].

We propose that temporal segregation of attraction and trapping is a more efficient strategy to maximize ant prey intake than a continuously low capture rate, particularly if recruitment leads to irregular, high peaks of ant density. If these coincide with a temporarily high capture rate, the overall prey intake would be higher than for a continuously low capture rate. The advantage of the temporal segregation strategy should be greatest for capturing ant species that use mass recruitment to exploit food resources. The attractiveness of a resource often increases nonlinearly with the number of individuals already exploiting, or recruiting to, that resource [17,29]. Temporary ineffectiveness of traps allows the plant to take advantage of this reinforcement effect: not only can more ants be recruited before the peristome is activated but the attractiveness of the trap is also more likely to persist for longer under wet conditions because more pheromone trails have been laid.

The exploitation of collective behaviour is not unique to *Nepenthes* but has a parallel in the behaviour of animal predators. Schooling, the formation of large coordinated groups in fish, has evolved as a strategy to reduce risks from predation [30]; however, this is exploited by some predators to maximize capture rates [31]. In both cases (ants and fish), the benefits of the collective behaviour are likely to exceed the losses through exploitation. For pitcher-visiting ants, the net energy gain from exploiting a nectar source that is highly abundant in space and time might outweigh the loss of workers from the colony [23].

The peristome with its wetness-based trapping mechanism is found almost ubiquitously in the genus *Nepenthes*. Ants attracted by nectar are the dominant prey not only in most *Nepenthes* species [13–15] but also in all other genera of pitcher plants [32–37], which often have strikingly similar trapping surfaces [2,38] and mechanisms [39] to those of *Nepenthes*. Hence, the ‘scout effect’ may represent a widespread ecological strategy and should be investigated in other genera of carnivorous plants.

## Supplementary Material

Figure S1

## Supplementary Material

Table S2

## Supplementary Material

Appendix S3

## Supplementary Material

Raw data S4

## Data accessibility

The raw data are available as electronic supplementary material S4.
