# Supplementary material for: How to catch more prey with less effective traps: explaining the evolution of temporarily inactive traps in carnivorous pitcher plants
Source: Proc Biol Sci. 2015 Feb 22;282(1801):20142675. doi: 10.1098/rspb.2014.2675 (PMC4309005; doi:10.1098/rspb.2014.2675)
Supplement: Figure S1 [file rspb20142675supp1.pdf]

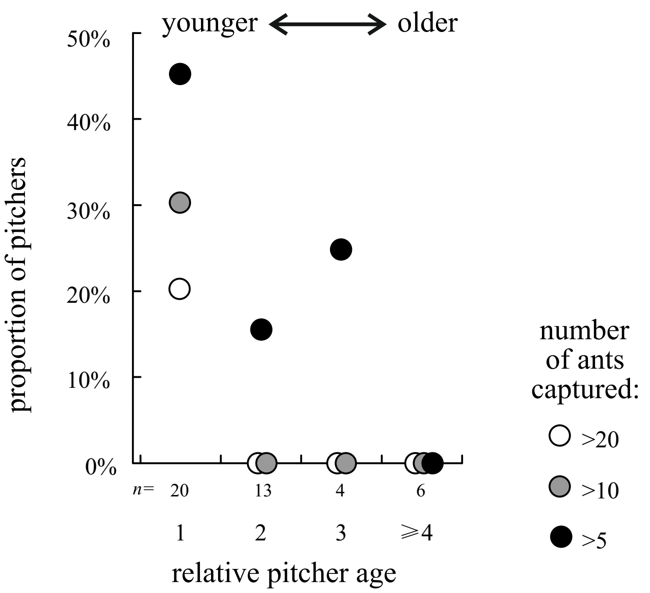

Figure S1. Frequency of batch capture events at three different thresholds (five, 10 and 20 ants of one species) in relation to (relative) pitcher age. Batch capture events, especially for larger batch sizes, occur mainly in the youngest pitchers on each shoot.
