# Supplementary material for: How to catch more prey with less effective traps: explaining the evolution of temporarily inactive traps in carnivorous pitcher plants
Source: Proc Biol Sci. 2015 Feb 22;282(1801):20142675. doi: 10.1098/rspb.2014.2675 (PMC4309005; doi:10.1098/rspb.2014.2675)
Supplement: Table S2 [file rspb20142675supp2.docx]

**Table S2.** Batch capture events were marginally more common in ‘lower’ than in ‘upper’ pitchers. However, this effect was not statistically significant.

|  | ‘Upper’ pitchers | ‘Lower’ pitchers |
| --- | --- | --- |
| *n* | 29 | 14 |
| Catches ≥20 | 1 | 3 |
| Catches ≥10 | 2 | 4 |
| Catches ≥5 | 7 | 5 |
