# Supplementary material for: How to catch more prey with less effective traps: explaining the evolution of temporarily inactive traps in carnivorous pitcher plants
Source: Proc Biol Sci. 2015 Feb 22;282(1801):20142675. doi: 10.1098/rspb.2014.2675 (PMC4309005; doi:10.1098/rspb.2014.2675)
Supplement: Appendix S3 [file rspb20142675supp3.docx]

**Appendix S3:**

**Optimality model for nutrient intake under continuous or intermittent trap activation**

This model explores the effectiveness of two alternative prey trapping strategies of pitchers (constant vs. intermittent trap activation) as a function of the recruitment strength of visiting ants and the relative importance of ants as prey. We consider two cases: linear and non-linear (sigmoidal) ant recruitment.

I. Linear recruitment

We assume that recruitment takes place only for ants and that the number of recruited ants increases linearly with the number of surviving visitors (scouts).

The ant visitor frequency *f_ants_* (visitors/time) is:

$f_{ants}=f_{ants}^{\left(0\right)}+f_{ants}^{\left(0\right)}\cdot r\left[1-E\right]=f_{ants}^{\left(0\right)}\left[1+r-rE\right]$ (1)

where *f_ants_^(0)^* is the baseline frequency without any recruitment (assumed to be the same under wet and dry conditions), *r* is the ants’ recruitment rate (dimensionless, defined as the number of recruited nestmates per scout), and *E* (dimensionless) denotes the capture probability, in this case for ants. We make the simplifying assumption that capture probability is equal for all types of prey.

The overall frequency of ant visitors will depend on the amount of recruitment occurring under wet and dry conditions. It is assumed here for the sake of simplicity that for a particular wetting condition, the frequency of ant visitors is constant, ignoring fluctuations and the effects of the detailed sequence of wet and dry times:

$\begin{matrix} f_{ants}=p_{wet}\cdot f_{ants}^{\left(0\right)}\left[1+r-rE_{wet}\right]+\left(1-p_{wet}\right)\cdot f_{ants}^{\left(0\right)}\left[1+r-rE_{dry}\right]= \\ =f_{ants}^{\left(0\right)}\left[1+r\left(1-E_{dry}+p_{wet}E_{dry}-p_{wet}E_{wet}\right)\right] \end{matrix}$ (2)

where *p_wet_* (dimensionless) is the proportion of the daytime when the peristome is wet ($p_{dry}=1-p_{wet}$).

The overall nutrient input *I* (mass/time) for the pitcher is:

$\begin{matrix} I=p_{wet}E_{wet}\left(f_{ants}m_{ants}+f_{other}m_{other}\right)+\left(1-p_{wet}\right)E_{dry}\left(f_{ants}m_{ants}+f_{other}m_{other}\right)= \\ =\left(f_{ants}m_{ants}+f_{other}m_{other}\right) \left(p_{wet}E_{wet}+\left(1-p_{wet}\right)E_{dry}\right) \end{matrix}$ (3)

where *m* is the mean nitrogen mass per prey, and *f_other_* and *m_other_* are the frequency and nitrogen mass for all other (non-recruiting) arthropods visiting the pitcher.

We now consider two possible scenarios, corresponding to alternative pitcher capture strategies: (a) traps are continuously active, and (b) visitors are only trapped when the peristome is wet.

*Scenario (a): traps continuously active*

In this case, the capture rate under wet and dry conditions is the same:

$E_{dry}=E_{wet}=E$ (4)

The nutrient input is:

$\begin{matrix} I=E\left(f_{ants}^{\left(0\right)}m_{ants}\left[1+r\left(1-E\right)\right]+f_{other}m_{other}\right)= \\ =-E^{2}f_{ants}^{\left(0\right)}m_{ants}r+E\left(f_{ants}^{\left(0\right)}m_{ants}+f_{ants}^{\left(0\right)}m_{ants}r+f_{other}m_{other}\right) \end{matrix}$ (5)

*I* is a negative quadratic function of *E*, which can have a maximum for

$\begin{matrix} \frac{dI}{dE}=-2Ef_{ants}^{\left(0\right)}m_{ants}r+f_{ants}^{\left(0\right)}m_{ants}\left(r+1\right)+f_{other}m_{other}=0 \\ E=\frac{f_{ants}^{\left(0\right)}m_{ants}\left(r+1\right)+f_{other}m_{other}}{2f_{ants}^{\left(0\right)}m_{ants}r}=\frac{r+1+\psi}{2r} \end{matrix}$ (6)

where $\psi=\frac{f_{other}m_{other}}{f_{ants}^{\left(0\right)}m_{ants}}$ denotes the abundance and nutritional importance of other insects relative to ants in the absence of recruitment.

Equation 6 shows that if traps were continuously active, a maximal capture rate (*E* = 1) would only be optimal if recruitment is weak or absent (*r* ≤ 1+*ψ*). For stronger recruitment (*r* > 1+*ψ*), lower (sub-maximal) capture rates would be better, as they promote scout survival. If other insects are more abundant than ants (high *ψ*), generally higher capture rates are favoured. These conclusions are consistent with previous predictions for pitcher plants, and with the increase in non-recruiting prey under constant wetting in this study [1,2].

*Scenario (b): visitors only trapped when peristome is wet*

The pitcher traps are active when wet (*E_wet_* > 0) but inactive when dry:

$\begin{matrix} E_{dry}=0 \\ f_{ants}=f_{ants}^{\left(0\right)}\left[1+r\left(1-E_{wet}p_{wet}\right)\right] \end{matrix}$ (7)

Now the nutrient input is:

$\begin{matrix} I=E_{wet}p_{wet}\left(f_{ants}^{\left(0\right)}m_{ants}\left[1+r\left(1-E_{wet}p_{wet}\right)\right]+f_{other}m_{other}\right)= \\ =-\left(E_{wet}p_{wet}\right)^{2}f_{ants}^{\left(0\right)}m_{ants}r+E_{wet}p_{wet}\left[f_{ants}^{\left(0\right)}m_{ants}+f_{ants}^{\left(0\right)}m_{ants}r+f_{other}m_{other}\right] \end{matrix}$ (8)

As shown for *E* in equation (6), *I* has a maximum at

$p_{wet}=\frac{r+1+\psi}{2E_{wet}r}$ (9)

Analogous to the conclusions for scenario (a), a continuous wetness (*p_wet_* = 1) would be best if recruitment is weak or absent ($r\leq\frac{1+\psi}{2E_{wet}-1}$). For stronger recruitment ($r>\frac{1+\psi}{2E_{wet}-1}$), intermittent wetness would be better, favouring scout survival. If other insects are more abundant than ants (high *ψ*), or for lower capture rates under wet conditions (0.5 < *E_wet_* <1), intermittent wetting would only be favoured for even stronger recruitment. If *E_wet_* < 0.5, intermittent wetting is never predicted to be optimal. As *p_wet_* > 0.5 for all possible values of *r, E_wet_* and *ψ*, the model suggests that the peristome should be wet for more than 12 hours per day. This is probably true for most *Nepenthes*, even those growing in open habitats [3].

The results are illustrated in Figure S3.1 and Figure 6 in the main text.

**Figure S3.1:** Predictions from the linear recruitment model (equation 9, plotted here for *E_we_*_t_ = 1) showing under which conditions continuous or varying degrees of intermittent wetting maximises the pitchers’ capture rate, depending on the recruitment rate of visiting ants and the relative importance of ants or other insects as prey. Intermittent wetting of pitchers (red) is favoured if ant recruitment is strong (high *r*) and if ants dominate the pitcher visitor spectrum (low *ψ*).

II. Non-linear (sigmoidal) recruitment

Non-linear recruitment in ants has been modelled previously using the following equation [4,5]:

$f_{ants}=\frac{f_{\max}\left(k+R\right)^{x}}{\left(k+R\right)^{x}+\left(f_{\max}-R\right)^{x}}$ (10)

*f_max_* is the maximum number of ants (per time) that can be recruited to a pitcher. The dimensionless parameter *x* determines the steepness of the sigmoidal recruitment curve, i.e. how strongly recruitment increases with the frequency of recruiters *R* (recruiters/time); *k* (units of inverse time) is a parameter that determines the attractiveness of the pitcher without any recruitment. The parameters *k* and *f_0_* depend on one another, as for *R*=0:

$f_{0}=\frac{f_{\max}}{1+\left(f_{\max}/k\right)^{x}} or k=f_{\max}\left(\frac{f_{0}}{f_{\max}-f_{0}}\right)^{\frac{1}{x}}$ (11)

The second term in the denominator has been adjusted to ensure *f_ants_ = f_max_* for *R= f_max_*. Transformation gives:

$f_{ants}=\frac{f_{\max}}{1+\left(\frac{f_{\max}-R}{k+R}\right)^{x}}=\frac{f_{\max}}{1+\left(\frac{f_{\max}-f_{0}\left[1-E\right]}{k+f_{0}\left[1-E\right]}\right)^{x}}=\frac{f_{\max}}{1+\left[\frac{1-\alpha\left[1-E\right]}{\left[\alpha/\left(1-\alpha\right)\right]^{1/x}+\alpha\left[1-E\right]}\right]^{x}}$ (12)

where $\alpha=f_{0}/f_{\max}$ is the dimensionless number of recruiters relative to the maximum.

The case *x* = 1 corresponds to linear recruitment:

$f_{ants}=\frac{f_{\max}\left(k+R\right)}{k+f_{\max}}=\frac{f_{\max}\left(\frac{f_{0}}{f_{\max}-f_{0}}\right)+R}{\left(\frac{f_{0}}{f_{\max}-f_{0}}\right)+1}=\frac{f_{\max}f_{0}+R\left(f_{\max}-f_{0}\right)}{f_{\max}}=f_{0}+rR$ (13)

where $r=1-f_{0}/f_{\max}$ is the recruitment rate as defined above.

Scenario (a): traps continuously active

Traps are continuously active and the nutrient input is:

$\begin{matrix} I=\frac{Ef_{\max}m_{ants}}{1+\left(\frac{1-\alpha\left[1-E\right]}{\left[\alpha/\left(1-\alpha\right)\right]^{1/x}+\alpha\left[1-E\right]}\right)^{x}}+Ef_{other}m_{other}= \\ =f_{\max}m_{ants}\left(\frac{E/\alpha}{1+\left(\frac{1-\alpha\left[1-E\right]}{\left[\alpha/\left(1-\alpha\right)\right]^{1/x}+\alpha\left[1-E\right]}\right)^{x}}+E\psi\right) \end{matrix}$ (14)

This function of *E* can have a maximum, depending on the parameters *ψ*, *α* and *x*. The conditions under which such a maximum exists for *E* < 1 can only be found numerically; the result is shown below.

**Figure S3.2:** Conditions (as predicted from the non-linear sigmoidal recruitment model) on the parameters *ψ* (importance of other insects in relation to ants), *α* (relative number of recruiters) and *x* (recruitment strength) for which the pitcher’s nutrient input is maximised by a sub-maximal capture rate (i.e. *E_opt_* < 1). For illustration, ant recruitment curves are plotted for several parameter combinations based on equation 10. A sub-maximal capture rate of pitchers is favoured (red) for intermediate numbers of recruiters (*α* ≈ 0.5) and strong recruitment (high *x*), both leading to a steep initial gradient of the recruitment curve. Moreover, a sub-maximal capture rate is favoured if ants dominate the pitcher visitor spectrum (low *ψ*).

The plot seems to suggest that lower capture rates are not favoured for linear recruitment (*x*= 1), contradicting the conclusions above. In this model, however, the recruitment rate is limited to $r=1-\alpha<1$, for which also the linear model above predicts continuous wetting to be optimal.

*Scenario (b): visitors only trapped when peristome is wet*

The traps are active when wet but inactive when dry. We assume here for simplicity that all visitors are trapped under wet conditions:

$\begin{matrix} E_{wet}=1, and E_{dry}=0 \\ R_{wet}=0 and R_{dry}=f_{0} \end{matrix}$ (15)

$f_{ants}=p_{wet}f_{0}+\frac{\left(1-p_{wet}\right)f_{\max}}{1+\left(\frac{1-\alpha}{\left(\alpha/\left(1-\alpha\right)\right)^{\frac{1}{x}}+\alpha}\right)^{x}}$ (16)

The overall nutrient input for the pitcher is:

$$I=p_{wet}^{2}m_{ants}f_{0}+\frac{p_{wet}\left(1-p_{wet}\right)m_{ants}f_{\max}}{1+\left(\frac{1-\alpha}{\left(\alpha/\left(1-\alpha\right)\right)^{\frac{1}{x}}+\alpha}\right)^{x}}+p_{wet}m_{other}f_{other}=$$

$=p_{wet}^{2}m_{ants}f_{0}-\frac{p_{wet}^{2}m_{ants}f_{\max}}{1+\left(\frac{1-\alpha}{\left(\alpha/\left(1-\alpha\right)\right)^{\frac{1}{x}}+\alpha}\right)^{x}}+p_{wet}m_{other}f_{other}+\frac{p_{wet}m_{ants}f_{\max}}{1+\left(\frac{1-\alpha}{\left(\alpha/\left(1-\alpha\right)\right)^{\frac{1}{x}}+\alpha}\right)^{x}}$ (17)

*I* is a negative quadratic function of *p_wet_*, which has a maximum for

$\frac{dI}{dp_{wet}}=\frac{-2p_{wet}m_{ants}f_{\max}}{1+\left(\frac{1-\alpha}{\left(\alpha/\left(1-\alpha\right)\right)^{\frac{1}{x}}+\alpha}\right)^{x}}+2p_{wet}m_{ants}f_{0}+m_{other}f_{other}+\frac{m_{ants}f_{\max}}{1+\left(\frac{1-\alpha}{\left(\alpha/\left(1-\alpha\right)\right)^{\frac{1}{x}}+\alpha}\right)^{x}}=0$ $p_{wet}=\frac{\left[\psi+\psi\left(\frac{1-\alpha}{\left(\alpha/\left(1-\alpha\right)\right)^{\frac{1}{x}}+\alpha}\right)^{x}+\frac{1}{\alpha}\right]}{2\left(\frac{1}{\alpha}-1-\left(\frac{1-\alpha}{\left(\alpha/\left(1-\alpha\right)\right)^{\frac{1}{x}}+\alpha}\right)^{x}\right)}$ (18)

Intermittent wetting is favoured if there exists a maximum for *p_wet_* < 1, which is shown here for *ψ* = 0.5,1 and 2 (Figure S3.3).

**Figure S3.3:** Conditions (as predicted from the non-linear sigmoidal recruitment model) on the parameters *ψ* (importance of other insects in relation to ants), *α* (relative number of recruiters) and *x* (recruitment strength) for which the pitcher’s nutrient input is maximised by intermittent wetting (i.e. *p_wet_* < 1). Ant recruitment curves are plotted for illustration as above. Intermittent wetting of pitchers is favoured (red) for small numbers of recruiters (0.1 < α < 0.4) and strong recruitment (high *x*), both increasing the gradient of the recruitment curve. Moreover, intermittent wetting is favoured if ants dominate the pitcher visitor spectrum (low *ψ*).

As for the linear recruitment model, *p_wet_* > 0.5 for all possible values of *α* and *ψ*. The longer the dry times of the day (corresponding to lower values of *p_wet_*), the higher the required recruitment strength (high *x* values, see Fig.S3.4)

**Figure S3.4:** Optimal proportion of wet times of the day (as predicted from the non-linear sigmoidal recruitment model), depending on *α* (relative number of recruiters) and x (recruitment strength), plotted here for *ψ* = 1 (equal nutritional importance of other insects and ants). Ant recruitment curves are plotted for illustration as above. Longer dry times (lower values of *p_wet_*) are favoured for small *α* and a high recruitment strength (high *x*).

**References**

[1] Joel DM. 1988 Mimicry and mutualism in carnivorous pitcher plants (Sarraceniaceae, Nepenthaceae, Cephalotaceae, Bromeliaceae). *Biol. J. Linn. Soc.* **35**, 185-197.

[2] Tan HTW. 1997 Prey. In *A guide to the carnivorous plants of Singapore* (ed. H. T. W. Tan), pp. 125-131. Singapore: Singapore Science Centre.

[3] Bauer U, Bohn HF, Federle W. 2008 Harmless nectar source or deadly trap: *Nepenthes* pitchers are activated by rain, condensation and nectar. *Proc. R. Soc. B* **275**, 259-265. (doi:10.1098/rspb.2007.1402)

[4] Shaffer Z, Sasaki T, Pratt SC. 2013 Linear recruitment leads to allocation and flexibility in collective foraging by ants. *Anim. Behav.* **86**, 967-975 (doi: 10.1016/j.anbehav.2013.08.014)

[5] Lanan MC, Dornhaus A, Jones EI, Waser A, Bronstein JL. 2012 The trail less traveled: individual decision-making and its effect on group behavior. *PLoS ONE* **7**, e47976. (doi: 10.1371/journal.pone.0047976)
