# Supplementary material for: How to catch more prey with less effective traps: explaining the evolution of temporarily inactive traps in carnivorous pitcher plants
Source: Proc Biol Sci. 2015 Feb 22;282(1801):20142675. doi: 10.1098/rspb.2014.2675 (PMC4309005; doi:10.1098/rspb.2014.2675)
Supplement: Raw data S4 [file rspb20142675supp4.pdf]

I. *Investigation of the natural prey capture mode*

Ants (1st/2nd sampling):

pitcher #	*IR*-1	*IR*-2	*CR*-1	*CR*-2	*CR*-3	MY-1	*TA*-1	*CA*-1	*POz*	*TE*-1	*TE*-2	UN-1	UN-2
UP-1-1-1	1/0	0/0	0/0	0/0	0/0	0/0	0/0	0/0	0/0	0/0	0/0	0/0	0/0
UP-1-2-1	0/0	0/0	0/2	1/1	0/0	0/0	0/0	0/1	0/0	0/2	0/0	0/0	0/0
UP-1-2-2	9/4	0/0	0/0	0/0	0/0	3/0	0/0	0/0	0/0	0/0	0/0	0/0	0/0
UP-1-2-3	5/6	0/0	0/0	0/0	0/0	0/0	0/0	0/0	0/0	0/0	0/0	0/0	0/0
UP-1-2-3	0/0	0/0	0/0	1/3	0/0	0/0	1/0	0/0	0/0	0/0	0/0	0/0	0/0
UP-1-3-1	1/0	0/0	0/0	0/0	0/0	0/0	0/0	0/0	0/0	0/0	0/0	0/0	0/0
UP-1-3-2	3/0	0/0	0/0	1/2	0/0	0/0	0/0	0/0	0/0	0/0	0/1	0/0	0/0
LP-1-4-1	0/0	0/0	0/0	16/5	0/0	5/5	19/4	3/3	0/0	0/0	0/0	1/0	0/0
LP-1-4-2	0/0	0/0	0/0	1/0	0/0	0/0	0/0	0/0	0/0	0/0	0/0	0/0	0/0
UP-2-1-1	0/-	0/-	0/-	0/-	0/0	2/-	0/-	0/-	0/-	0/-	0/-	0/-	0/-
UP-2-1-2	0/1	0/0	0/0	3/0	0/0	0/0	0/0	0/0	0/1	0/0	0/0	0/0	0/0
UP-2-1-3	0/0	0/0	0/1	0/0	0/0	4/0	0/0	0/0	0/0	0/0	0/0	0/0	0/0
UP-2-1-4	0/0	0/0	0/0	0/0	0/0	0/0	1/0	0/0	0/0	0/0	0/0	0/0	0/0
LP-2-2-1	0/0	0/0	0/0	0/0	0/0	3/2	2/7	0/0	0/0	0/0	0/0	0/0	0/0
LP-2-3-1	0/0	0/0	0/0	0/0	0/0	20/11	0/0	0/0	0/0	0/0	0/0	0/0	0/0
LP-2-3-2	0/0	0/0	0/0	0/0	0/0	0/0	0/0	0/0	0/0	0/0	0/0	0/0	0/0
LP-2-4-1	-/0	-/0	-/0	-/0	-/1	-/0	-/0	-/0	-/0	-/0	-/0	-/0	-/0
UP-3-1-1	2/0	0/0	0/1	0/0	0/0	4/1	0/0	0/0	0/0	0/0	0/0	0/0	0/0
UP-3-1-2	0/0	0/0	0/0	0/0	0/0	1/0	0/0	0/0	0/0	0/0	0/0	0/0	0/0
UP-3-2-1	0/0	0/0	0/0	0/0	0/0	7/1	0/0	0/0	0/0	0/0	0/0	0/0	0/0
UP-3-2-2	0/0	0/0	0/0	0/0	0/0	0/0	0/0	0/0	0/0	0/0	0/0	0/0	0/0
UP-3-3-1	0/0	0/0	0/0	0/0	0/0	6/1	0/0	0/0	1/0	0/0	0/0	0/0	0/0
UP-3-3-2	0/0	0/0	0/0	0/0	0/0	5/1	0/0	0/0	0/0	0/0	0/0	0/0	0/0
IP-3-4-1	3/0	0/0	0/0	0/0	0/0	12/1	0/0	0/0	1/0	0/0	0/0	0/0	0/0
LP-3-5-1	0/0	37/18	0/0	0/0	0/0	0/1	0/0	0/0	0/0	0/0	0/0	0/0	3/0
LP-3-6-1	1/0	31/7	0/0	0/0	0/0	0/0	0/0	0/0	0/0	0/0	0/0	0/0	0/0
LP-3-6-2	0/0	0/0	0/0	0/0	0/0	0/0	0/0	0/0	0/0	0/0	0/0	0/0	0/0
UP-4-1-1	0/0	0/0	0/0	0/0	0/0	2/0	0/0	0/0	0/0	0/0	0/0	0/0	0/0
UP-4-1-2	0/0	0/0	0/0	0/0	0/0	0/0	0/0	0/0	0/0	0/0	0/0	0/0	0/0
UP-4-2-1	0/0	0/0	0/0	0/0	0/0	0/1	0/0	0/0	0/0	0/0	0/0	0/0	0/0
UP-4-3-1	0/0	0/0	0/0	0/0	0/0	22/1	0/0	0/0	0/0	0/0	0/0	0/0	0/0
UP-4-3-2	0/0	0/0	0/0	0/0	0/0	1/0	0/0	0/0	0/0	0/0	0/0	0/0	0/0
UP-4-3-3	0/0	0/0	0/0	0/0	0/0	0/0	0/0	0/0	0/0	0/0	0/0	0/0	0/0
UP-4-3-4	0/0	0/0	0/0	0/0	0/0	0/0	0/0	0/0	0/0	0/0	0/0	0/0	0/0
UP-4-3-5	0/0	0/0	0/0	0/0	0/0	0/0	0/0	0/0	0/0	0/0	0/0	0/0	0/0
UP-4-3-6	0/0	0/0	0/0	0/0	0/0	0/0	0/0	0/0	0/0	0/0	0/0	0/0	0/0
UP-4-4-1	-/0	-/0	-/0	-/0	-/0	-/0	-/0	-/0	-/0	-/0	-/0	-/0	-/0
UP-4-4-2	0/0	0/0	0/0	0/0	0/0	0/0	0/0	0/0	0/0	0/0	0/0	0/0	0/0
LP-4-5-1	-/0	-/1	-/0	-/0	-/0	-/0	-/0	-/0	-/0	-/0	-/0	-/0	-/0
LP-4-5-2	0/0	0/0	4/0	0/0	0/0	3/1	0/0	0/0	0/0	0/0	0/0	0/0	0/0
LP-4-5-3	0/0	0/0	0/0	0/0	0/0	3/0	1/0	0/0	0/0	0/0	0/0	0/0	0/0
LP-4-5-4	0/0	0/0	0/0	0/0	0/0	0/0	0/0	0/0	0/0	0/0	0/0	0/0	0/0
LP-4-6-1	0/0	0/0	0/0	0/0	0/0	0/0	0/0	0/0	0/0	0/0	0/0	0/0	0/0

Pitchers are numbered as type-plant-shoot-pitcher, e.g. LP-4-5-1 is the youngest 'lower' pitcher on shoot #5 of plant #4.
Numbers in each cell are prey numbers for the 1st/2nd sampling. '-' denotes missing data.

Abbreviations: UP = 'upper' pitcher; LP = 'lower pitcher'; IP = intermediate pitcher; *IR* = *Iridomyrmex* msp.; *CR* = *Crematogaster* msp.; MY = Myrmicinae msp.; *TA* = *Tapinoma* msp.; *CA* = *Camponotus* msp.; *POz* = *Polyrhachis zopyra*; *TE* = *Tetraponera* msp.; UN = unidentified msp.

Other prey (1st/2nd sampling):

pitcher #	NA-1	ME-1	ME-2	MU	DI	CP	CM	LE	UFI	AR	CU	VE	HY	BL	AP	OR
UP-1-1-1	0/0	0/0	0/0	0/0	1/0	0/0	0/0	0/0	0/0	0/0	0/0	0/0	0/0	0/0	0/0	0/0
UP-1-2-1	0/0	0/0	0/0	0/0	0/0	0/0	1/0	0/0	0/0	0/0	0/0	0/0	0/0	0/0	0/0	0/0
UP-1-2-2	0/0	0/0	0/1	1/0	2/0	1/1	0/0	0/0	0/0	0/0	0/0	0/0	0/0	0/0	0/0	0/0
UP-1-2-3	0/0	0/0	0/0	1/0	0/0	1/0	0/0	0/0	1/0	0/0	0/0	0/0	0/0	0/0	0/0	0/0
UP-1-2-3	0/0	0/0	0/0	0/0	1/0	0/0	2/0	1/0	1/0	0/0	0/0	0/0	0/0	0/0	0/0	0/0
UP-1-3-1	0/0	0/0	0/0	0/0	0/0	0/0	0/0	0/0	0/0	0/0	0/0	0/0	0/1	0/0	0/0	0/0
UP-1-3-2	0/0	0/0	0/0	0/0	1/0	0/0	0/0	0/0	0/0	0/0	0/0	0/0	0/0	0/0	0/0	0/0
LP-1-4-1	0/0	0/0	0/0	0/0	0/0	0/0	1/0	0/0	1/0	0/0	0/0	0/0	0/0	0/0	0/0	0/0
LP-1-4-2	0/0	0/0	0/0	0/0	0/0	0/0	1/0	0/0	0/0	0/0	0/0	0/0	0/0	0/0	0/0	0/0
UP-2-1-1	0/-	0/-	0/-	1/-	3/-	1/-	2/-	0/-	0/-	0/-	0/-	0/-	0/-	0/-	0/-	0/-
UP-2-1-2	0/0	1/0	0/0	2/0	6/0	0/0	0/0	0/0	0/0	0/0	0/0	0/0	0/0	0/0	0/0	0/0
UP-2-1-3	0/0	0/0	0/0	0/0	4/0	2/0	0/0	0/0	0/0	0/0	0/0	1/0	0/0	0/0	0/0	0/0
UP-2-1-4	0/0	1/0	0/1	0/0	3/0	0/0	0/0	0/0	0/0	0/0	0/0	0/0	0/0	0/0	0/0	0/0
LP-2-2-1	5/0	0/0	0/0	0/0	0/0	0/0	0/0	0/0	0/0	0/0	0/0	0/0	0/0	0/0	0/0	0/0
LP-2-3-1	0/0	1/0	0/0	0/0	0/0	0/0	0/0	0/0	0/0	1/0	0/0	0/0	0/0	0/0	0/0	0/0
LP-2-3-2	1/0	0/0	0/0	0/0	0/1	0/0	0/0	0/0	0/0	0/0	0/0	0/0	0/0	0/0	0/0	0/0
LP-2-4-1	-/0	-/0	-/0	-/0	-/0	-/0	-/0	-/0	-/0	-/0	-/0	-/0	-/0	-/0	-/0	-/0
UP-3-1-1	0/0	0/0	0/1	0/0	2/1	1/1	0/0	0/0	0/0	0/1	0/0	0/0	0/0	0/0	0/0	0/0
UP-3-1-2	0/0	0/0	0/1	0/0	5/0	2/0	0/0	0/0	0/0	0/0	0/0	0/0	1/0	0/0	0/0	0/0
UP-3-2-1	0/0	0/0	0/0	0/0	5/0	2/1	0/0	1/0	0/0	0/0	0/0	0/0	0/0	0/0	0/0	0/0
UP-3-2-2	0/0	0/0	0/0	1/2	3/0	1/0	0/0	0/0	0/0	0/0	0/0	0/0	0/0	0/0	0/1	0/0
UP-3-3-1	0/0	0/0	0/0	1/0	1/0	0/0	0/0	0/0	0/0	0/0	0/0	0/0	0/0	0/0	0/0	0/0
UP-3-3-2	0/0	0/0	0/0	0/0	1/0	0/0	0/0	0/0	0/0	0/0	0/0	0/0	0/0	0/0	0/0	0/0
IP-3-4-1	0/0	0/0	0/0	0/0	0/0	1/0	0/0	0/0	0/0	0/0	1/0	0/0	0/0	0/0	0/0	0/0
LP-3-5-1	86/0	0/0	0/0	0/0	0/0	0/0	0/0	0/0	0/0	0/0	0/0	0/0	0/0	0/0	0/0	0/0
LP-3-6-1	0/0	0/0	0/0	0/0	0/0	0/0	0/0	0/0	0/0	0/0	0/0	0/0	0/0	0/0	0/0	0/1
LP-3-6-2	0/0	0/0	0/0	0/0	0/0	0/0	0/0	0/0	0/0	0/0	0/0	0/0	0/0	0/0	0/0	0/0
UP-4-1-1	0/0	0/0	0/0	1/0	4/0	0/0	1/0	0/2	0/0	0/0	0/0	0/0	0/0	0/0	0/0	0/0
UP-4-1-2	0/0	0/0	0/0	0/0	2/0	0/0	0/0	0/0	0/0	0/0	0/0	0/0	0/0	0/0	0/0	0/0
UP-4-2-1	0/0	0/0	0/0	0/0	0/1	0/0	0/0	0/0	0/0	0/0	0/0	0/0	0/0	0/0	0/0	0/0
UP-4-3-1	0/0	3/0	1/2	0/0	9/1	3/0	0/0	0/0	0/0	0/0	0/0	0/0	0/0	0/0	0/0	0/0
UP-4-3-2	0/0	0/0	1/0	0/0	4/1	2/1	0/0	0/0	0/0	0/0	0/0	0/0	0/0	0/0	0/0	0/0
UP-4-3-3	0/0	0/0	0/0	0/0	4/1	0/1	0/0	0/0	0/0	0/0	0/0	0/0	0/0	0/0	0/0	0/0
UP-4-3-4	0/0	0/0	0/0	0/0	0/0	0/0	0/0	1/0	0/1	0/0	0/0	0/0	0/0	0/0	0/0	0/0
UP-4-3-5	0/0	0/0	0/0	0/0	1/0	1/0	0/0	0/0	0/0	0/0	0/0	0/0	0/0	0/0	0/0	0/0
UP-4-3-6	0/0	0/0	0/0	0/0	0/0	1/0	0/0	0/0	0/0	0/0	0/0	0/0	0/0	0/0	0/0	0/0
UP-4-4-1	-/0	-/0	-/0	-/0	-/0	-/0	-/0	-/0	-/0	-/0	-/0	-/0	-/0	-/0	-/0	-/0
UP-4-4-2	0/0	0/0	0/0	0/0	0/0	0/0	0/0	0/0	0/1	0/0	0/0	0/0	0/0	0/0	0/0	0/0
LP-4-5-1	-/48	-/0	-/0	-/0	-/0	-/0	-/0	-/0	0/1	-/0	-/0	-/0	-/0	-/0	-/0	-/0
LP-4-5-2	0/0	0/0	0/0	0/0	0/0	0/0	0/0	0/0	1/0	1/0	0/0	0/0	0/0	0/0	0/0	0/0
LP-4-5-3	0/0	0/0	0/0	0/0	0/0	0/0	0/0	0/0	0/0	1/0	0/0	0/0	0/0	1/0	0/0	0/0
LP-4-5-4	0/0	0/0	0/0	0/0	0/0	0/0	0/0	0/0	0/0	0/0	0/0	0/0	0/0	0/0	0/0	0/0
LP-4-6-1	0/0	0/0	0/0	0/0	0/0	0/0	0/0	0/0	0/0	0/0	0/0	0/0	0/0	0/0	0/0	0/0

Abbreviations: NA = Nasutitermitinae; ME = Meliponini msp.; MU = Muscidae; DI = unidentified Diptera; CP = Coleoptera; CM = Collembola; LE = Lepidoptera; UFI = unidentified flying insects; AR = Araneae; CU = Curculionidae; VE = Vespidae; HY = unidentified Hymenoptera; BL = Blattodea; AP = Apidae; OR = Orthoptera

II. *Comparison of contiuously vs. intermittently active traps: prey capture*

Ants:

	Experimental period I (A = wetted; B = control)					Experimental period II (A = control; B = wetted)				
**pitcher #**	**S1**	**S2**	**S3**	**S4**	**S5**	**S1**	**S2**	**S3**	**S4**	**S5**
1A	5	0	1	1	2	0	0	2	1	0
1B	5	5	4	1	0	4	0	0	0	0
2A	0	4	10	3	0	0	3	16	132	5
2B	0	5	10	1	4	0	0	0	1	0
3A	0	0	0	0	1	0	0	1	0	0
3B	0	0	0	0	0	0	0	0	0	0
4A	0	1	0	0	0	0	0	0	0	0
4B	0	0	0	0	0	0	0	1	0	1
5A	1	0	0	0	0	0	0	0	1	0
5B	0	1	0	0	0	0	0	0	0	0
6A	3	0	3	0	0	0	0	0	0	0
6B	3	3	19	1	1	1	5	1	0	1
7A	0	0	1	1	0	0	0	1	0	0
7B	2	1	0	0	0	3	0	0	0	0
8A	0	0	0	0	0	0	0	0	0	0
8B	0	0	0	0	0	0	0	0	0	0
9A	0	0	2	0	0	0	0	0	2	0
9B	0	0	0	0	0	0	0	0	0	0
10A	0	0	1	0	0	0	0	1	0	0
10B	0	0	0	0	0	0	0	0	0	0
11A	0	0	0	0	0	0	0	0	-	-
11B	0	0	0	0	0	0	0	1	-	-

Numbers are prey counts per sample; '-' denotes missing data. A and B pitchers for each number are on the same plant. Experimental conditions (wetted, control) were exchanged between A and B pitchers at the end of experimental period I.

Abbreviations: S = sample

S3 and S4 were following 2-day intervals, all others were sampled daily.

Flying prey:

pitcher #	Experimental period I (A = wetted; B = control)					Experimental period II (A = control; B = wetted)				
	S1	S2	S3	S4	S5	S1	S2	S3	S4	S5
1A	0	0	2	1	0	0	0	2	0	0
1B	1	1	2	0	0	0	0	10	1	0
2A	1	2	2	0	0	0	0	3	0	0
2B	1	0	0	1	0	0	0	5	0	0
3A	0	3	1	3	0	1	0	6	2	0
3B	0	1	2	0	0	2	2	5	1	1
4A	3	1	1	2	0	0	0	2	2	2
4B	3	0	1	1	0	1	0	2	2	1
5A	0	2	0	2	0	0	1	2	1	0
5B	0	1	0	0	0	0	0	0	3	0
6A	0	0	0	0	1	0	0	2	0	0
6B	0	0	2	1	1	0	0	0	2	0
7A	1	0	1	0	0	0	1	1	0	0
7B	1	0	0	0	1	0	0	4	0	0
8A	0	1	5	0	0	1	1	1	0	0
8B	1	1	1	0	1	0	0	1	1	1
9A	1	2	2	1	0	0	0	1	0	0
9B	1	1	2	0	0	0	0	7	3	1
10A	0	0	0	1	0	0	0	2	0	0
10B	0	0	1	0	0	0	0	1	0	0
11A	0	2	2	0	0	0	0	1	-	-
11B	0	2	0	0	0	0	0	3	-	-

III. *Comparison of contiuously vs. intermittently active traps: visitor numbers*

Ants:

pitcher #	condition	Time after trail disruption (h)									
		1	2	3	4	5	6	7	8	9	10
1	wetted	0	0	5	8	4	12	8	8	4	14
2	control	0	0	0	0	0	1	1	0	1	0
3	wetted	0	0	0	0	0	0	0	0	0	0
4	control	0	0	0	0	0	0	0	1	1	1
5	control	0	0	0	0	0	0	0	0	0	0
6	wetted	1	0	0	0	0	0	0	1	0	0
7	control	0	0	0	0	0	2	5	2	3	2
8	wetted	2	0	0	0	0	0	0	0	0	0
9	control	1	0	0	0	0	0	0	0	3	0
10	wetted	0	0	0	0	0	1	1	0	0	0
11	control	0	3	9	10	17	25	20	13	21	22
12	wetted	0	0	1	1	1	2	2	0	0	2
13	control	0	0	1	2	0	0	0	0	0	0
14	wetted	1	2	3	0	0	0	2	7	8	10
15	control	0	0	0	0	0	0	0	0	0	2
16	wetted	1	1	5	2	8	2	3	0	2	0
17	control	0	1	3	0	0	0	0	1	4	2
18	wetted	0	0	0	1	0	1	0	0	0	0
19	control	0	0	0	0	0	0	0	0	0	0

Other visitors:

pitcher #	condition	Time after trail disruption (h) 1	2	3	4	5	6	7	8	9	10
1	wetted	3	0	0	0	0	0	0	3	0	0
2	control	3	2	0	0	0	0	0	0	0	0
3	wetted	0	0	1	0	1	0	3	0	0	0
4	control	0	4	0	0	1	0	2	0	0	1
5	control	0	2	1	0	0	0	1	0	0	1
6	wetted	1	1	1	0	0	0	0	0	3	0
7	control	0	0	1	0	1	1	0	0	0	0
8	wetted	1	0	0	1	0	0	0	0	0	1
9	control	1	1	1	1	0	1	0	0	0	1
10	wetted	0	0	1	0	0	1	0	0	0	0
11	control	3	1	1	0	0	0	0	0	3	4
12	wetted	0	0	0	0	1	1	1	0	0	1
13	control	3	1	3	0	1	0	3	3	0	1
14	wetted	0	3	1	0	0	1	0	0	1	0
15	control	0	0	0	0	0	0	0	1	2	0
16	wetted	1	0	0	0	0	0	0	0	0	0
17	control	4	2	0	0	0	1	4	0	1	2
18	wetted	1	0	0	0	2	0	0	0	0	0
19	control	0	0	0	0	0	0	2	3	1	3

Visitor numbers are pooled for each hour. Each pitcher was observed on a different day.
Observations were made with a digital camera taking a photograph every 5 minutes.
